# PEGylated PLGA-based phase shift nanodroplets combined with focused ultrasound for blood brain barrier opening in rats

**DOI:** 10.18632/oncotarget.17155

**Published:** 2017-04-17

**Authors:** Xiang Zhang, Jiangang Hu, Guanjian Zhao, Ning Huang, Ying Tan, Li Pi, Qing Huang, Feng Wang, Zhigang Wang, Zhibiao Wang, Yuan Cheng

**Affiliations:** ^1^ Department of Neurosurgery, The Second Affiliated Hospital of Chongqing Medical University, Chongqing, 400010, China; ^2^ Chongqing Key Laboratory of Ultrasound Molecular Imaging, Institute of Ultrasound Imaging of Chongqing Medical University, Chongqing, 400016, China; ^3^ Chongqing Key Laboratory of Biomedical Engineering, College of Biomedical Engineering of Chongqing Medical University, Chongqing, 400016, China

**Keywords:** blood brain barrier, focused ultrasound, acoustic droplet vaporization, perfluoropentane, PLGA

## Abstract

Previous studies have shown that focused ultrasound (FUS) combined with systematic administration of microbubbles (MBs) can open the blood brain barrier (BBB) locally, transiently and reversibly. However, because of the micro size diameters, MBs are restricted in the intravascular space and cannot extravasate into diseased sites through the opened BBB. In this study, we fabricated one kind of nanoscale droplets which consisted of encapsulated liquid perfluoropentane cores and poly (ethyleneglycol) - poly (lactide-co-glycolic acid) shells. The nanodroplets had the capacity to realize liquid to gas phase shift under FUS. Significant extravasation of Evan's blue appeared when acoustic pressure reached 1.0 MPa. Intracerebral hemorrhages and erythrocyte extravasations were observed when the pressure was increased to 1.5 MPa. Prolonged sonication duration could enhance the level of BBB opening and broaden the time window simultaneously. Furthermore, compared with MBs, the distribution of EB extravasation was firmly confined within narrow region in the center of focal zone, suggesting the site of FUS induced BBB opening could be controlled with high precision by this procedure. Our results show the feasibility of serving PEGylated PLGA-based phase shift nanodroplet as an effective alternative mediating agent for FUS induced BBB opening.

## INTRODUCTION

Blood-brain barrier (BBB) is the foremost obstacle which highly prevents effective therapeutic agents from transferring into brain parenchyma and functioning properly. For achieving an effective therapeutic concentration at diseased tissues, the doses of drugs that cannot cross BBB must be high enough. However, high drug doses can easily induce severe side-effects and undesired drug accumulation at non-diseased sites. Therefore, studies aimed at providing helpful ways for drug delivery across BBB with high efficiency are necessary. It has been demonstrated that focused ultrasound (FUS) could locally, transiently and reversibly increase the permeability of BBB in the presence of microbubbles(MBs), which shows tremendous potentials for targeted delivery of chemotherapeutic agents [1–3], antibodies [4, 5], genes [6, 7], cells [8] and so on.

Conventional commercial or self-made MBs consist of inert gaseous cores and monolayer lipid shells which prevent fusion of bubbles and dissolution of gas into the surrounding medium. Under proper ultrasound pressure, MBs oscillate regularly and the repetitive contraction and expansion of MBs create microstreamings in nearby medium. This is known as stable cavitation [3, 9]. The microstreamings could apply shear forces to the cerebrovascular endothelial cells and increase the cell permeability. When the pressure exceeds a certain level, MBs will collapse and generate forceful shock waves and microjets (called inertial cavitation). These powerful stresses can create pores within the cell membrane, break tight junctions between the adjacent brain endothelial cells, and finally enhance the transcellular and paracellular permeability of BBB [10]. Although these hypotheses are supported by convincing experimental data, the exact mechanism of FUS induced BBB opening in the presence of MBs is still unclear.

Because of the micron size diameters, the remaining MBs in circulation after sonication are too bulky to extravasate into brain parenchyma through the opened pathway. Additionally, MBs only have a circulation half-life of seconds to minutes [11] that re-administration during procedure is generally needed. Therefore, a new kind of mediating agent with smaller size and longer circulation half-life for FUS induced BBB opening is necessary.

Phase shift perfluorocarbon droplet, which generally consists of a liquid perfluorocarbon (PFC) core and a coating shell, can be excited by ultrasound and vaporize into gas bubbles if the acoustic power is high enough. This phenomenon is called acoustic droplet vaporization (ADV) [12–15]. The threshold for vaporization is increased with decreasing diameter and decreased with increasing ultrasound frequency and sonication time [13, 16]. A predominant factor for successful vaporization of PFC droplets is the acoustic pressure [17, 18]. Theoretically, as long as circulating through the route of FUS beam, the disturbance for MBs induced by ultrasound energy will happen. It might lead to undesired cavitation effects at non-target area. But for phase shift droplets, the disturbance will occur only when the pressure is high enough to initiate ADV. This characteristic ensures the cavitation effects is limited in a stricter region and provides a promise for ultra-precise control of BBB opening. Moreover, the diameter of droplet can be easily manufactured into nanoscale size, so the residual non-excited nanodroplets have the potential to extravasate into brain parenchyma through opened BBB.

The aim of this study was to synthesize one kind of polymeric phase shift perfluorocarbon nanodroplets and investigated whether it can serve as an alternative mediating agent for FUS induced BBB opening. Due to the applicable natural boiling point (29^°^C), perfluoropentane (PFP) was chosen in this study. To achieve this aim, PEGylated PLGA-based PFP-encapsulated (PEG-PLGA-PFP) nanodroplets were prepared by a double emulsion method. Poly (ethylene glycol) - poly (lactide-co- glycolic acid) (PEG-PLGA), an amphiphilic copolymer which contained merits of PEG for long half-time and high stability in circulation [19, 20] and PLGA for good biocompatibility and biodegradability [21, 22], was selected as the shell materials.

## RESULTS

### Characterization and thermal evaporation of PEG-PLGA-PFP nanodroplets

**Figure 1:**
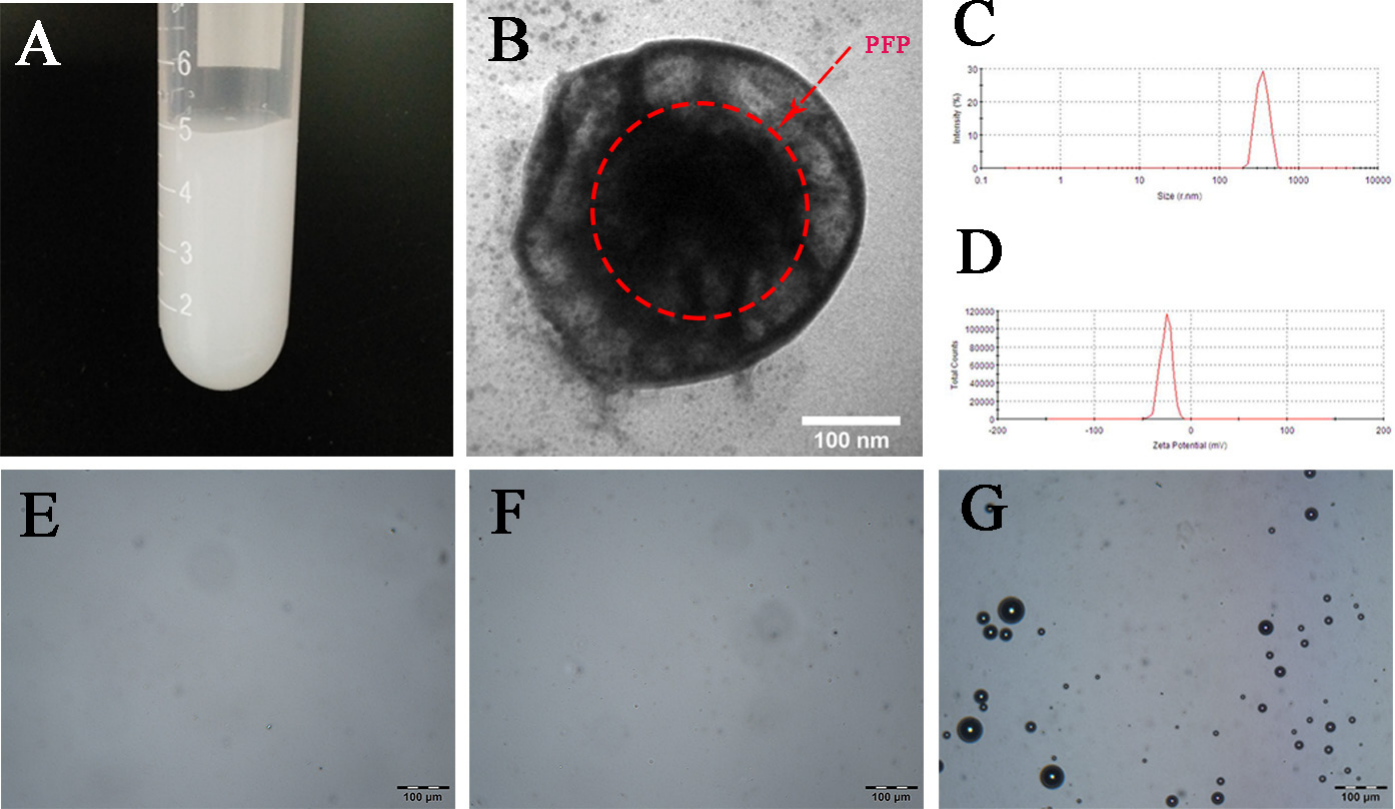
Characterization and *in vitro* thermal evaporation of PEG-PLGA-PFP nanodroplets (**A**) A digital image of PEG-PLGA-PFP nanodroplets suspension (5 mg/ml). (**B**) A TEM image of PEG-PLGA-PFP nanodroplets. The red dotted circle shows the PFP inner core. (**C** and **D**) Size distribution and zeta potential of PEG-PLGA-PFP nanodroplets. (**E**–**G**) Microscope images(×200) of PEG-PLGA-PFP nanodroplets heated to 29^°^C (E), 37^°^C (F) and 48^°^C (G), bar:100 μm.

The PEG-PLGA-PFP nanodroplets suspension (5 mg/ml) was shown ivory (Figure 1A). Transmission electron microscope (TEM) image (Figure 1B) revealed the spherical shell-core morphology of PEG-PLGA-PFP nanodroplet and the inner center dark mass of the sphere indicated the existence of liquid PFP core. The mean size distribution was 316.0 ± 7.9 nm (Figure 1C) with a polydispersity index of, and the mean zeta potential was −26.3 ± 0.9 mV (Figure 1D). The acoustic stability of nanodroplet suspension for 5-day storage at 4^°^C had been checked (Supplementary Figure 1). The nanodroplets suspension (1 mg/ml) remained to be non-echo for two days after fabrication, but a very small amount of strong echoes came out three days after fabrication. As time went on, more and more strong echoes came out along with increasing of gray scale value. For preventing invalidation, therefore, PEG-PLGA-PFP nanodroplets should be used within two days after fabrication.

To further confirm the successful encapsulation of PFP and detected the liquid to gas phase shift temperature, PEG-PLGA-PFP nanodroplets were heated from room temperature (20^°^C) to 55^°^C. As shown in Figure 1E, 1F, no obvious bubbles were generated when the temperature reached 29^°^C and 37^°^C. After the temperature reached 48^°^C (Figure 1G), numerous of bubbles were generated which indicated the successful encapsulation of PFP. The paradoxical phenomenon that why the phase shift temperature was higher than the natural boiling point of PFP is attributed to the changes of interfacial surface tension [23, 24]. As the decrease of diameter into nano size, the boiling point of PFP was raised by the increasing partial pressure within the droplet. This characteristic also guaranteed the biostability of PEG-PLGA-PFP nanodroplets in circulation at physiological temperatures [25].

### FUS-induced phase shift *in vitro*

**Figure 2:**
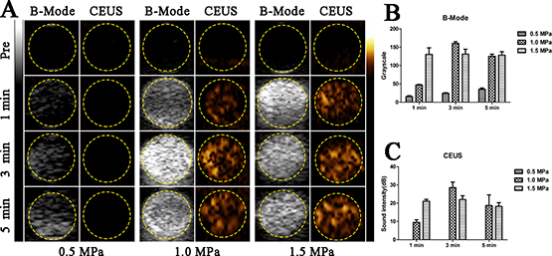
*In vitro* FUS-induced ADV of PEG-PLGA-PFP nanodroplets (**A**) B-Mode and CEUS images of nanodroplets before and after FUS sonication. (**B** and **C**) The quantitative analysis of grayscales in B-Mode (B) and acoustic intensities in CEUS (C) of nanodroplets after FUS sonication. (*n* = 3).

Figure 2A illustrates the B-Mode and contrast-enhanced ultrasound (CEUS) images of PEG-PLGA-PFP nanodroplets suspension (1 mg/ml) before and after FUS sonication at an acoustic pressure of 0.5MPa, 1.0MPa or 1.5MPa and sonication duration of 1 min, 3 min or 5 min. Before FUS sonication, the B-Mode images showed PEG-PLGA-PFP nanodroplets were anechoic and no contrast-enhanced signals were detected. When acoustic pressure reached 1.0MPa, the B-mode and CEUS images showed noticeably increased echogenicity and contrast enhancement which indicated the successful phase shift and generation of microbubbles. The histograms showed the quantitative analysis of mean gray scale (Figure 2B) and mean acoustic intensity value (Figure 2C). Comparatively, the B-Mode and CEUS images were the strongest at acoustic pressure of 1.0MPa and sonication duration of 3 min (Table 1).

**Table 1:** Value of gray scale and acoustic intensity of PEG-PLGA-PFP nanodroplets suspension after FUS sonication

Pressure (MPa)	Duration (min)	Gray scale	Acousitc intensity (dB)
0.5	1	15.4 ± 2.4	0
0.5	3	24.4 ± 1.5	0
0.5	5	35.6 ± 3.2	0
1.0	1	47.0 ± 1.6	9.5 ± 1.4
1.0	3	160.4 ± 4.6	28.6 ± 3.0
1.0	5	125 ± 6.1	18.7 ± 5.8
1.5	1	130.6 ± 17.8	21.1 ± 1.0
1.5	3	131.0 ± 13.5	22.0 ± 2.1
1.5	5	128.4 ± 9.3	18.3 ± 2.0

(*n* = 3).

### Evan's blue extravasation

**Figure 3:**
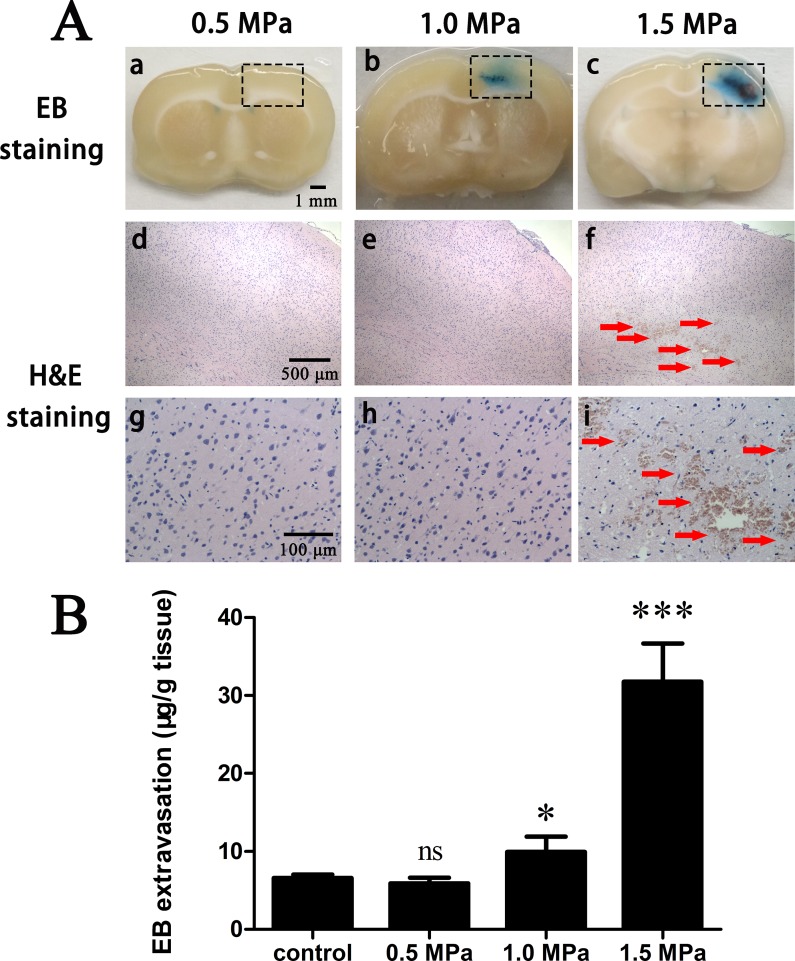
EB extravasation and H&E stain (**A**) FUS combined with PEG-PLGA-PFP nanodrplets induced BBB opening with acoustic pressure of 0.5 MPa, 1.0 MPa and 1.5 MPa. Significant EB extravasation was realized at acoustic pressure 1.0 MPa (b) and increasing pressure would enhance the level (c). Intracerebral hemorrhages and sporadic erythrocyte extravasations (f, i) appeared at 1.5 MPa. Black rectangle: FUS sonication area. Upper row (a-c): gross brain section; middle row (d–g): enlarged (×40) corresponding black rectangle region in H&E stain; bottom row (g–i): enlarged (×200) corresponding black rectangle region in H&E stain. Red arrow: Intracerebral hemorrhages and sporadic erythrocyte extravasations. (**B**) Quantitative analysis of EB extravasation *in vivo* after FUS sonication combined with PEG-PLGA-PFP nanodroplets at an acoustic pressure of 0.5 MPa, 1.0 MPa or 1.5 MPa with sonication duration of 3 min. (ns = not significant,*p < 0.05, ***p < 0.001 as compared with the control, *n* = 5).

The feasibility of *in vivo* BBB opening via FUS combined with PEG-PLGA-PFP nanodroplets was verified by qualitatively and quantitatively assessing extravasations of Evan's Blue (EB) in the sonicated side (right cerebral hemisphere) compared with the control (left cerebral hemisphere). The upper row of Figure 3A showed the images of coronal brain sections and the EB extravasations indicated the area of BBB opening via FUS combined with nanodroplets. Figure 3B illustrates the quantitative analysis of the amount of EB extravasations in the sonicated side at an acoustic pressure of 0.5 MPa, 1.0 MPa or 1.5 MPa with duration of 3min. At 0.5MPa, the quantified EB extravasation showed no statistical significance compared with control and no brain tissues were stained by EB. When the acoustic pressure was increased to 1.0MPa, the statistical significance was achieved (*P* < 0.05) and a very clear EB extravasation area within the right brain appeared. In the center of the extravasation area we could see a nearly 2mm long but very narrow region which had a significant deep blue compared with the surrounding tissue. The long-narrow area was perpendicular to the FUS pathway and we believed this area demonstrated where BBB was successfully and intensively opened. As the acoustic pressure was increased to 1.5MPa, the quantified EB extravasations showed a significant increase and the central deep blue area apparently became longer and a little wider. Thus, 1.0MPa could be considered as the acoustic pressure threshold for FUS-induced BBB opening combined with PEG-PLGA-PFP nanodroplets in this study.

### Histological evaluation

H&E stain (Figure 3A, middle and bottom rows) was performed in order to assess the safety of this procedure. No erythrocyte extravasations or hemorrhages were observed at 0.5 MPa and 1.0 MPa. When acoustic pressure was increased to 1.5 MPa, both the regions of BBB opening and the EB extravasation levels were significantly enhanced, but were accompanied by intracerebral hemorrhages and sporadic erythrocyte extravasations. These damages to the brain could be explained by the powerful inertial cavitation that disrupted the tight junctions too excessively in the focal region. Thus, 1.0 MPa may be a practicable acoustic pressure at which BBB could be successfully opening and biosecurity was satisfied at once, and we chose acoustic pressure of 1.0 MPa to investigate the time window.

### Time window

**Figure 4:**
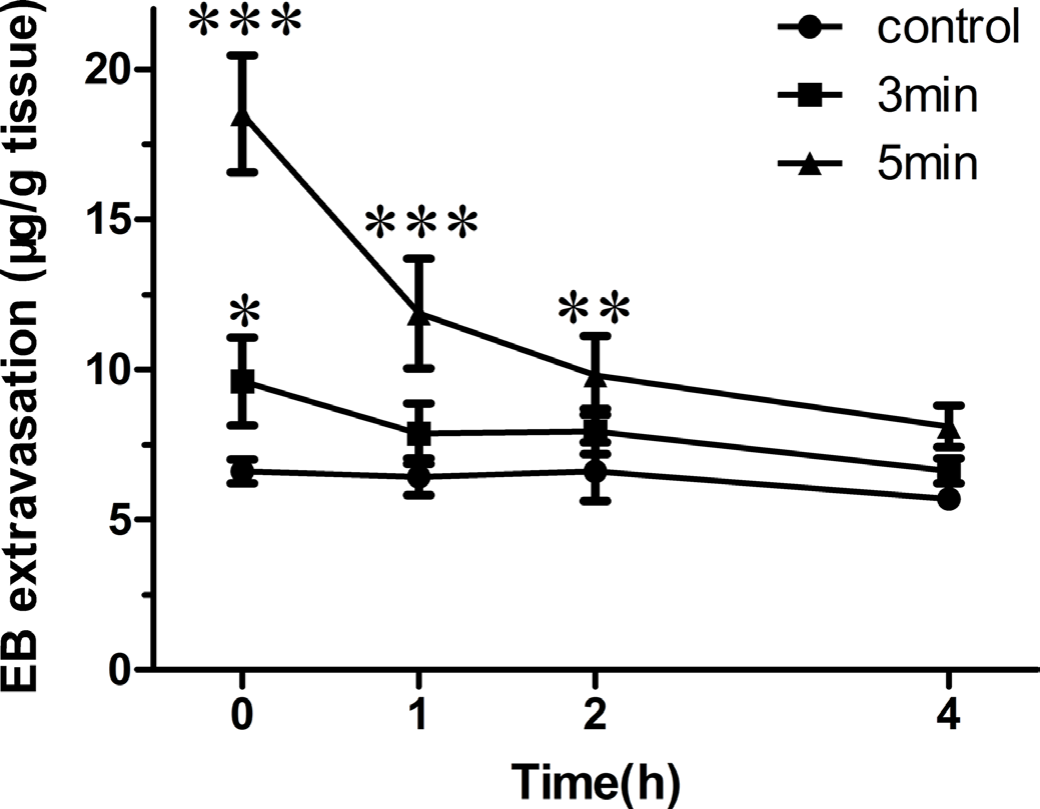
EB extravasation in the brain tissue was quantified 0, 1, 2 or 4 h after 1.0 MPa sonication with duration of 3 or 5 min (*p < 0.05, **p < 0.01, ***p < 0.001 as compared with the control, *n* = 3).

Figure 4 demonstrates the amounts of EB extravasations changed as a function of time in the sonicated side at an acoustic pressure of 1.0MPa and sonication duration of 3 or 5 min. EB extravasation reached the highest at beginning, and prolonged sonication duration could obviously enhance it. Then the values showed continuous declining which indicated the re-establishment of BBB integrity, especially in the first hour. At the time point of 1 hour, because no statistical difference was achieved as compared to the control, the re-establishment of BBB was seemed to be accomplished for brains underwent a 3 min sonication. For the other group, the time window was extended to 4 hours. Thus, increased sonication duration could enhance the level and prolong the time window of BBB opening.

### The difference of EB extravasation

**Figure 5:**
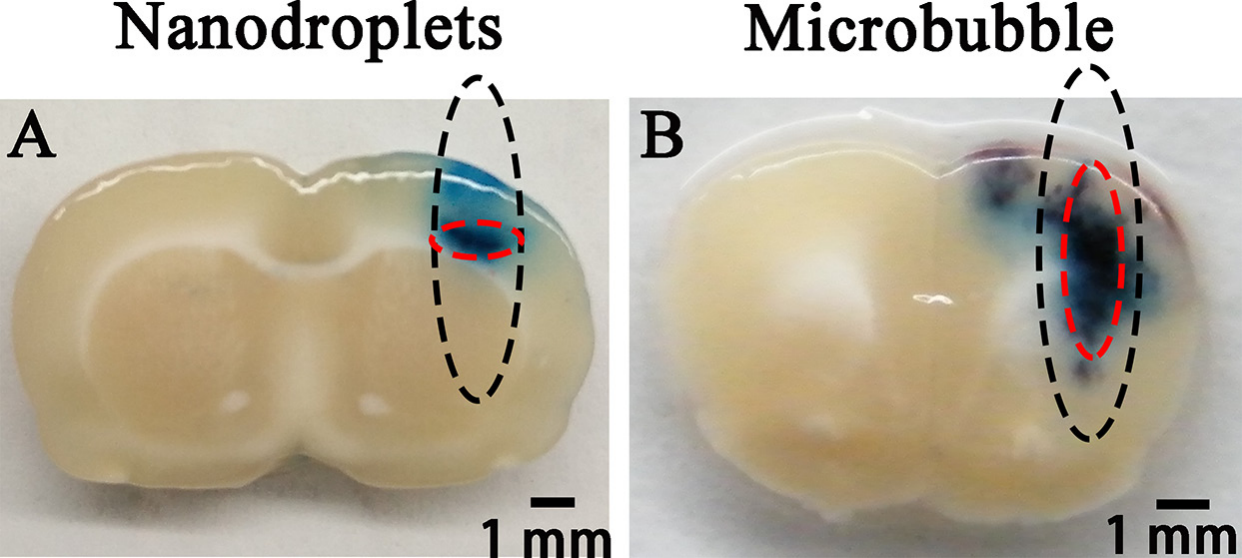
Distribution pattern of EB extravasation when PEG-PLGA-PFP nanodroplets (**A**) or lipid MBs (**B**) was used as mediating agent combined with FUS. The black dotted circles show the half-maximum of the pressure amplitude of the focal zone. The red dotted circles show the deep blue region of EB extravasation which indicates the location of BBB opening.

Figure 5 shows the difference of distribution pattern of EB extravasation induced by FUS combined with PEG-PLGA-PFP nanodroplets (Figure 5A) or lipid-shelled MBs (Figure 5B). Both the deep blue regions were mostly distributed in the half-maximum of the pressure amplitude of the focal zone. For MBs, the long axis of the EB extravasation distribution followed the route of FUS beam, which was similar to our previous studies [6, 26]. For PEG-PLGA-PFP nanodroplets, however, the long axis was perpendicular to the route, and the region of EB extravasation was firmly restricted in the central narrow area of the focal zone. Even when the acoustic power was increased to 1.5 MPa at which severe brain injuries appeared, the minor axis of the region just became a little wider (Figure 4A).

## DISCUSSION

FUS combined with lipid-shelled MBs has been verified as an effective and powerful tool for overcoming the barrier function of BBB at present. The main purpose of this study was to investigate whether PEGylated PLGA-based PFP-contained phase shift nanodroplets could be used to replace MBs as a novel mediating agent, and attempted to find out the difference of BBB opening regions between these two. In fact, this is not the first study that used phase shift nanodroplets as the mediating agent for FUS induced BBB opening. Chen et al. [27] had done an excellent job on it. They first generated one kind of lipid-shelled MBs in which contained perfluorobutane (PFB) gaseous cores, and then condensed the MBs into nanodroplets. However, due to the low boiling point (−2^°^C) of PFB, they had to adopted relatively low temperature and high pressure for condensation. It raised demands on production conditions for other researchers. But the process provided by us could be performed just in an ice bath under normal atmospheric pressure. In the meantime, compared with lipid, the degradation process of PLGA can be regulated by adjusting the molecular weight or the ratio of lactide to glycolic acid (LA/GA). Increasing the molecular weight of PLGA will prolong the degradation time and raising the proportion of LA in PLGA will slow the degradation rate. These characteristics make PLGA an ideal skeleton material for drug delivery system [28].

The qualitative and quantitative analysis of EB extravasation confirmed the successful BBB opening via FUS combined with PEG-PLGA-PFP nanodroplets when the acoustic pressure reached 1.0MPa. It was bigger than the threshold (0.45MPa) that Chen et al. achieved. The main reason could be PFP has a higher boiling point (29^°^C) so more pressure is needed. Secondly, the PLGA-based shell is more unyielding than lipid-based shell, so more power is required to break the shell down. Distributions of EB extravasation induced by nanodroplets were heavily limited in the central area of the focal zone, even when the pressure was increased to 1.5 MPa at which intracerebral hemorrhages and erythrocyte extravasations appeared. For MBs, on the other hand, the EB extravasation appeared along the pathway of FUS in the focal zone. This difference between PEG-PLGA-PFP nanodroplets and MBs confirmed the theory that phase shift nanodroplets could only be excited when the acoustic pressure is high enough to excite it transforms from ground state (nanodroplet) to excited state (microbubble). This will be useful for high accuracy control of FUS induced BBB opening. Besides, the unscheduled second BBB opening area owing to the sound reflection on the skull base may be avoided. To our best knowledge, this is the second study to use phase shift nanodroplets as the mediating agents for FUS induced BBB opening and the first to use PLGA and PFP as essential ingredients. Interestingly and notablely, Airan et al. [29] had just described a novel technology that could silence seizures in an acute rat seizure model. They fabricated PFP-encapsulated phase shift polymeric nanoparticle carriers of propofol. The carriers were excited by transcranial MR-guided focused ultrasound (tcMRgFUS) sonication. And the propofol, which is a small enough to cross BBB, was targeted released during the liquid to gas phase transition. But the MRI data showed no evidence of BBB opening so that the brain homeostasis was maintained during the procedure. These two studies by Airan et al. and us are complementary, indicating that we might be about to reach the critical line. More studies on this are necessary.

Several major shortcomings of this study should be pointed out. Frist of all, a skull bone window was still a need. The energy attenuation induced by the presence of the skull seriously hindered the application of this technology. Second, lack of powerful guiding system made us can hardly focus on a uniform site of the brain. The tcMRgFUS has been proved to be an attractive and effective non-invasive technology for central nervous system diseases, such as seizure [29], trigeminal neuralgia [30], essential tremor [31], centrally located brain tumours [32], and Parkinson's disease [33, 34]. Thus, using tcMRgFUS may remedy these two defects at once. Shen et al. [35] demonstrated the successful delivery of liposomes with different sizes (55 nm, 120 nm and 200 nm) through opened BBB after FUS sonication (1.282 MHz + 0.53 or 0.64 MPa + 60 s) in the presence of microbubbles. The nanodroplets we fabricated seemed to be too large. But as researches continue and technics advance, we believe that nanodroplets could one day successfully cross the opened BBB. The last and most serious shortcoming is the tremendously weak comparability of the compare, and the different materials used for fabrication makes it difficult to improve. The reason why we want to carry out this compare was due to the lack of studies on the difference of distribution pattern of BBB opening between nanoparticles and MBs. The results seemed to be positive for enhancing focusing performance by using phase shift nanodroplets. More high-quality comparisons are needed.

PLGA nanoparticles have been proved to be one kind of effective drug delivery system [36–39]. For the nanodroplets that we composed in this study, hydrophobic therapeutic agents can be easily encapsulated in the PLGA shell [40], and further surface modification with specific ligands would make these nanodroplets have active-targeting function [38]. Ho et al. [41] demonstrated that drug penetration can be enhanced via vascular disruption induced by using phase shift nanoscale droplets. Thus, drug loaded targeted phase shift nanodroplets combined with FUS for simultaneous BBB opening and extravascular therapy in the brain would be the next main direction for future study.

## MATERIALS AND METHODS

### Materials

PEG-PLGA (5000/12000, lactide/glycolic acid = 50/50) copolymer was purchase from the Shan-dong Key Laboratory of Medical Polymer Materials (Shan-dong, China). PFP was purchased from Alfa Aesar (U.K.). Poly (vinyl alcohol) (PVA, 99%, MW = 30,000–70,000 Da) and EB were obtained from Sigma-Aldrich Corp. (St. Louis, MO, USA). Self-made lipid-shelled MBs was provided by Guanjian Zhao. All chemicals used in this work were of analytical grade and were used as received.

### Preparation of PEG-PLGA-PFP nanodroplets

Phase shift nanodroplets were prepared by a modified double emulsion/solvent evaporation method. Briefly, 50 mg of PEG-PLGA was dissolved by 3 ml dichloromethane (DCM) in a glass tube. The tube was inserted in the ice during dissolution. When PEG-PLGA was fully dissolved, 0.4 ml of liquid PFP was added to the cold mixture and emulsified with an ultrasonic probe (SONICS & MATERIALS, Inc., USA) (100 w, 5 s on/5 s off, 1 min total). Once the sonication finished, 8 ml of cold PVA (4%) was added into the first emulsion immediately and carried out the second sonication (100 w, 5 s on/5 s off, 2 min total). To evaporate DCM, the resulting solution was diluted with 20 ml of cold PVA (4%) and stirred gently for 6 h. All of the processes mentioned above were carried out in ice bath. Subsequently, the final solution was centrifuged at 12,000 rpm for 8 min at 4^°^C. The supernatant was discarded and the precipitate was washed by deionized water. The process of centrifugation and washing was repeated three times. Finally, the nanodroplets were collected and stored at 4^°^C.

### Characteristics of PEG-PLGA-PFP nanodroplets

The size distribution and zeta potential of the nanodroplets were determined using a Malvern Zetasizer Nano ZS unit (Malvern Instruments, UK). For each sample, the mean diameter of three determinations was calculated. Values reported were the mean ± standard deviation of at least three different batches of nanodroplets. The morphology of PEG-PLGA-PFP nanodroplets was observed by TEM (Hitachi H-7600, Japan). To estimate the successful encapsulation of PFP, PEG-PLGA-PFP nanodroplets (1 mg/ml) were heated by a heating panel with a viewing hole and visualized under an inverted microscope.

### *In vitro* FUS-induced phase shift

A portable FUS system was provided by the Department of Biomedical Engineering of Chongqing Medical University. Continuous FUS exposures were generated by a 1 MHz single-element focused transducer (diameter = 40 mm, focus length = 18 mm). The diameter and length of the focal zone with half-maximum of the pressure amplitude were 2.2 mm and 6.9 mm, respectively. The transducer was fixed in a handheld probe with a panhead and the center of the focal zone was about 8.2 mm away from the tip.

**Figure 6:**
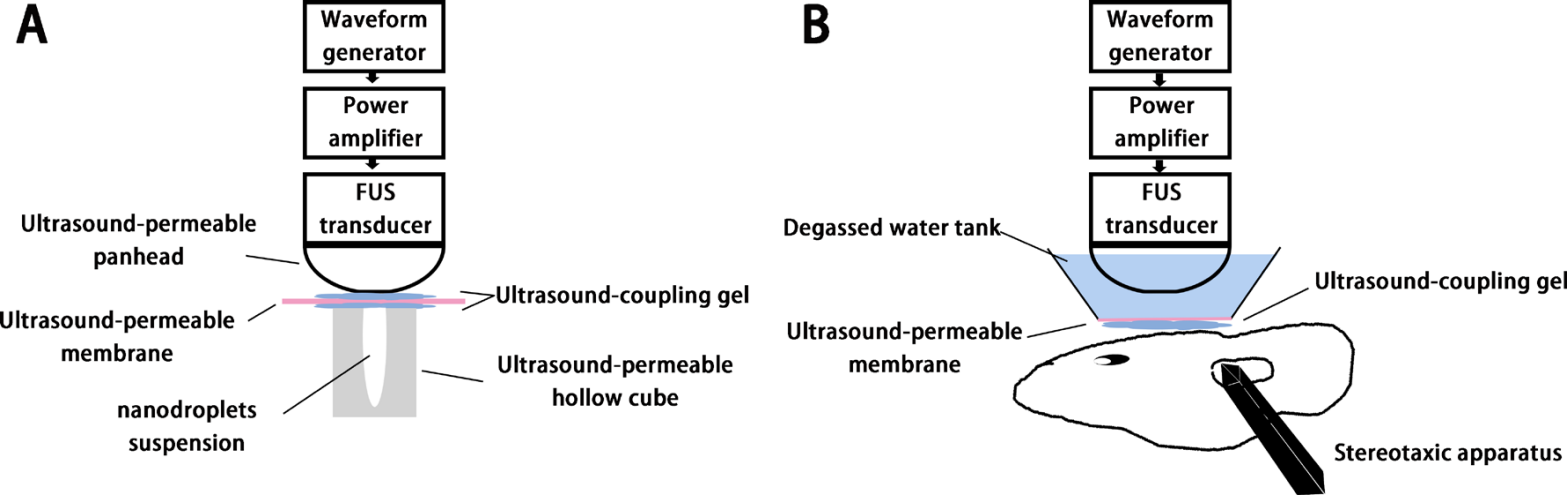
*In vitro* (**A**) and *in vivo* (**B**) experimental setup.

Figure 6A illustrated experimental setup for testing *in vitro* FUS-induced phase shift. Approximately 1 ml(1 mg/ml) of the PEG-PLGA-PFP nanodroplets suspension was injected into a hollow gel cuboid with strong sound transmission. Then the cuboid filled with nanodroplets underwent a FUS sonication along the long axis. The acoustic pressures ranged from 0.5 MPa to 1.5 MPa and the sonication time ranged from 1 min to 5 min. Cross sectional B-Mode ultrasonography and contrast-enhanced ultrasonography (CEUS) were used to detect whether phase shift was realized after sonication. MyLab 90 (Esaote, Italy) with a linear probe (5–12 MHz) was used as the ultrasonic imaging system for observing and capturing, and experiments were repeated three times. The mean gray scale and sound intensity (dB) of the nanodroplets suspension were then analyzed by the software DFY (invented by the Institution of Ultrasound Imaging of Chongqing Medical University, Chongqing, China) for further quantitatively analysis.

### Animal preparation

All procedures involving animals were approved by the Institutional Animal Care and Use Committee of Chongqing medical university. Male Sprague-Dawley (SD) rats weighing from 200 g to 250 g were used. The animals were housed in an IVC experimental animal house in which the temperature was maintained at 24^°^C. Before sonication, each animal was anesthetized intraperitoneally with chloral hydrate (400 mg/kg) and then inserted a catheter (24G; Deltaflo, Italy) in the tail vein for injections. The fur on the scalp was removed with an electric clipper and depilatory cream. The head was fixed on a stereotaxic apparatus (ZS Dichuang, China) and the body temperature was maintained at 37^°^C using a heating pad. A window (4 mm × 5 mm) at right parietal bone was opened for each animal and the right coronal suture crossed it along the middle line.

### *In vivo* experimental setup

Figure 6B illustrated experimental setup for testing *in vivo* FUS-induced BBB opening. The same FUS system was used for *in vivo* protocols. For relatively precise targeting, its handheld probe was fixed on the left-right axis of the stereotaxic apparatus, and the targeted region was set at the position of 3.0 mm lateral and 0.5 mm anterior to the bregma, and 3.0 mm below the skull surface. The space between probe and skull surface was fill with a self-made degassed water tank whose bottom was an ultrasound permeable membrane, and ultrasound coupling gel was applied between the skull and the membrane.

### Confirmation of BBB opening and assessment of brain tissue injury

Twenty-four rats were randomly divided into three groups. Continuous FUS sonication (0.5, 1.0 or 1.5 MPa) was performed 20s after intravenous administration of PEG-PLGA-PFP nanodroplets (10 mg/kg) into the tail vein, and the duration was fixed at 3 min. To verify the successful opening of BBB, rats were intravenously injected with EB (100 mg/kg) after FUS sonication immediately. Animals were sacrificed 4 h after the EB injection, and 0.9% normal saline was perfused into the left cardiac ventricle continuously until clear perfusion fluid flowed out of the right atrium. The brains were removed and sliced coronally after perfusion.

Five samples of each group were random selected, then divided into right and left hemispheres. The unsonicated left hemispheres were regarded as the control. Samples were weighed and then soaked in 50% trichloroacetic acid solution. After homogenization and centrfugation, the extracted dye was diluted with ethanol (1:3), and the amount present was measured using a spectrophotometer at 620 nm. The EB tissue content was quantifed via a linear regression standard curve derived from five concentrations of the dye and was denoted in terms of the amount per gram of tissue.

The rest three samples of each group were prepared for histological observation. Samples were fixed in 4% paraformaldehyde for 48 h. After post fixation processing, the sections were embedded in paraffin, sectioned at 5 μm thickness, and stained with hematoxylin and eosin (H&E). Histologic evaluation was performed by an author who was blind to the parameters of sonication.

### Time window

Twenty-four rats were were randomly divided into two groups. Continuous FUS sonication with duration of 3 or 5 min at 1.0MPa was performed 20s after intravenous administration of PEG-PLGA-PFP nanodroplets (10 mg/kg) into the tail vein. At 4 time points (0, 1, 2 or 4 h) after sonication, three rats of each group were selected for procedure of EB extravasation evaluation.

### Distribution pattern of EB extravasation

For investigating the difference of distribution pattern of EB extravasation, we used another three SD rats underwent a continuous FUS sonication combined with conventional self-made lipid MBs. The MBs were provided by Guanjian Zhao in our group. The concentration of MBs was adjusted to 10^8^/ml and the dose used for each rat was set at 2 × 10^8^/kg. The acoustic pressure was set at 1.0 MPa, and the sonication duration was set at 40 sec according to our previous study [6, 26].

### Statistical analysis

An unpaired student's *t* test was used for Statistical analysis and a probability (P) less than 0.05 was considered significant. Results were expressed as mean standard ± deviation (SD).

## CONCLUSIONS

In this study, PEGylated PLGA-based PFP-encapsulated phase shift nanodroplets were successfully fabricated. Significant EB extravasation and satisfied biosecurity could be achieved by the systematic administration of this nanodroplets combined with FUS at acoustic pressure of 1.0MPa. Prolonged duration of sonication could broaden the time window of BBB opening. In summary, these promising results indicate that the PEG-PLGA-PFP nanodroplets could serve as an effective alternative mediating agent and have great potential for ultra-precise control of BBB opening and target drug delivery.

## SUPPLEMENTARY FIGURE


