# Supplementary material for: PEGylated PLGA-based phase shift nanodroplets combined with focused ultrasound for blood brain barrier opening in rats
Source: Oncotarget. 2017 Apr 17;8(24):38927–36. doi: 10.18632/oncotarget.17155 (PMC5503583; doi:10.18632/oncotarget.17155)
Supplement: Supplementary file 1 [file oncotarget-08-38927-s001.pdf]

PEGylated PLGA-based phase shift nanodroplets combined with focused ultrasound for blood brain barrier opening in rats

Supplementary Materials

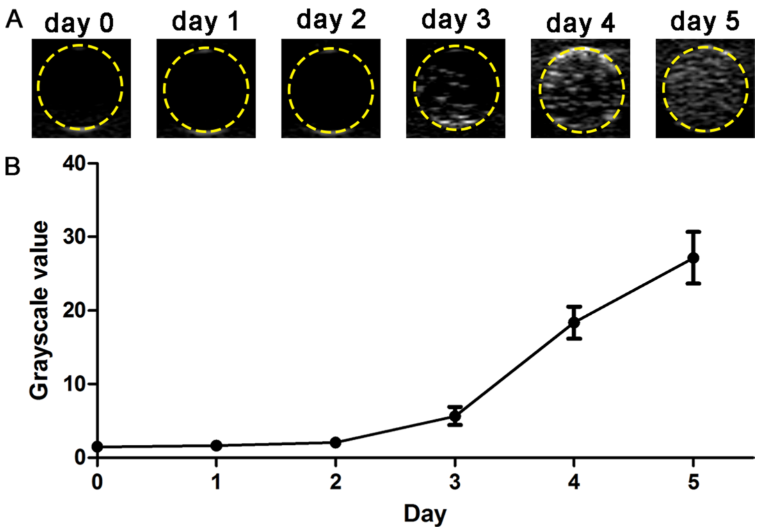

Supplementary Figure 1: Qualitative and quantitative analysis of acoustic stability of PEG-PLGA-PFP nanodrplets during 5-day storage at 4°C. (**A**) B-Mode images of nanodroplets at different time points after fabrication. (**B**) Quantitative analysis of gray scale value. (n = 3).

MATERIALS AND METHODS

Acoustic stability of nanodroplets

Eighteen ml fresh nanodroplets suspension (1 mg/ml) were divided into Eighteen EP tubes (1 ml for each) and stored at 4°C. Three of tubes were selected at different time points after fabrication (0, 1, 2, 3, 4 and 5 day), and B-Mode ultrasonography and quantitatively analysis of grayscale value were performed for each sample.
